# Comparative analysis of physiological responses to environmental stress in *Hedysarum scoparium* and *Caragana korshinskii* seedlings due to roots exposure

**DOI:** 10.7717/peerj.14905

**Published:** 2023-02-28

**Authors:** Juanli Ma, Huan Wang, Lei Jin, Ping Zhang

**Affiliations:** 1 School of Agriculture, Ningxia University, Yinchuan, Ningxia, China; 2 Chabuchaer County Forestry and Grassland Adminstraton, Ili Valley, Xinjiang, China

**Keywords:** Root exposure, Physiological index, Growth index, Photosynthetic index

## Abstract

*Hedysarum scoparium* and *Caragana korshinskii* seedlings were used as the test materials to carry out the roots exposure stress model test. By comparing the physiological growth indexes in the leaves of the tested plants, the stress resistance ability was evaluated. The results showed (1) root exposure resulted in excessive oxygen free radicals, which resulted in membrane lipid peroxidation and increased MDA content in two plants. The increase of MDA content in *H. scoparium* was greater than that in *C. korshinskii*. (2) *H. scoparium* mainly regulate their own adaptation to the stress by regulating carotenoids. *C. korshinskii* adjusts itself to adapt to the stress by regulating chlorophyll. (3) *H. scoparium* resist this stress mainly by regulating their respiration rate. (4) *H. scoparium* mainly through the mobilization of proline, by adjusting the proline concentration to reduce their water potential; *C. korshinskii* reduced its water potential mainly by regulating the concentration of soluble sugar to adapt to the stress. (5) *H. scoparium* and *C. korshinskii* activated peroxidase (*H. scoparium*) and catalase (*C. korshinskii*) to clean up intracellular peroxides, respectively. To sum up, under the same root exposure ratio, there were significant differences in physiological regulation and morphological indexes between *H*. and *C. korshinskii*, but their stress resistance mechanisms were quite different.

## Introduction

There are many deserts and sandy lands in northwest China. The commonly used artificial fixation method for convective dunes is planting grass square grid and interplanting shrub. *Hedysarum scoparium* and *Caragana korshinskii* were commonly used as sand-fixing plant species on moving and semi-moving dunes. The fixation method of mobile dune usually planted sand-fixing plants such as *H. scoparium* and *C. korshinskii* in grass square. However, when the grass square sand barrier failed, plants in the active area of wind-blown sand would be exposed to the roots by wind erosion. Plant roots can not only fix the above-ground parts of plants, but also are essential organs that absorb nutrients and water from the soil (Cochavi, Cohen & Rachmilevitch, 2020; Gruber et al., 2013), so the root is a key factor affecting the average, expected growth of plants (Stattin, Hellqvist & Lindström, 2000). In the early stage of sand fixation, the stability of the sand surface was poor, and the seedlings had weak resistance. After the seedlings were exposed to wind erosion, their roots would suffer from freezing damage, high temperature, and mechanical damage (Xianji et al., 2017).

Drought has a significant impact on plant physiology and biochemistry (Hsiao, 1973), it inhibis the growth of plants by disrupting the dynamic balance of reactive oxygen species, changing the solute in cell compatibility, inhibiting photosynthesis, and reducing water productivity (Farooq et al., 2009; Lipiec et al., 2013; Duan et al., 2018; Rohit et al., 2016; Moller Ian, Erik & Andreas, 2007; Zadworny et al., 2021). At the same time, plants would experience a series of survival reactions, such as the use of stomatal regulation, osmotic regulation (Kosar et al., 2021; Raja et al., 2020; Kaya et al., 2020; Zhu, 2016), and antioxidant defense, *etc*. (Apel & Hirt, 2004; Zhang et al., 2018; Ahmad et al., 2010; Ahmad et al., 2019; Kohli et al., 2019). Under low temperatures in winter and night conditions, roots, seedlings, and other organs or cells would be frozen and dehydrated (Weiser, 1970; Uemura & Steponkus, 1994; Grossnickle & South, 2014). The shoots might have frost resistance, while the roots might not have good frost resistance (Stattin, Hellqvist & Lindström, 2000). Studies on high-temperature stress (Koh, 2002) had shown that short-term exposure of plants to a temperature of 38–40 °C would cause the transient expression of heat-shock proteins(HSPs), causing a “high heat-shock response.” After the roots of sand plants were exposed, the erosion of sand would cause different degrees of mechanical damage to their roots. Mechanical damage was also significant for plants. Although plants could release some substances from damaged cells to repair the broken parts for minor injuries (Heil & Land, 2014; Toyota et al., 2018; Li et al., 2020), for severe injuries, plants would die if they can not repair themselves.

Therefore, revealing the physiological and biochemical regulation mechanism of wind erosion resistance of sand-fixing plants, such as *H. scoparium* and *C. korshinskii*, had a guiding significance for the selection and breeding of wind-proof and sand-fixing plants in the future.

## Materials and Methods

### Experimental design

The experiment lasted for 2 months, from November 15, 2018, to January 19, 2019. It was conducted in the greenhouse of the agricultural science training base of Ningxia University. To facilitate the exposure of the root system, a 28 cm × 30 cm (diameter × height) non-woven bag was used for seedling cultivation. The seeds of *H. scoparium* and *C. korshinskii* were washed before sowing, then soaked in warm water at 22 °C, and kept in a thermostat for 24 h. When sowing, sand was packed up to 5 cm at the mouth of the bag and watered to moist (Sand could be squeezed into a ball with hand, but water would not overflow). The seeds were evenly spotted on the sand surface and covered with a layer of sand twice as thick as the seeds. Afterward, they were watered quantitatively every 4 days, and the soil moisture in the sandbags was measured before and after treatment (Table 1). To ensure that the control soil moisture of each treatment was not significantly different from the control group, the soil moisture of 6 cm *C. korshinskii* root exposed was considerably lower than other treatments. Still, the soil moisture was more than 3.7% (The soil moisture of *C. korshinskii* in Baijitan Nature Reserve is usually between 3% and 4%), which would not cause severe drought stress to the plant.

**Table 1:** Growth indicators of two shrubs after root exposure. The table shows the changes of growth indexes of *Hedysarum scoparium* and *Caragana korshinskii* under different root exposure treatments.

Growth indexes	*Hedysarum scoparium*				*Caragana korshinskii*			
Root exposure length ratio	0 L	1/4 L	1/2 L	3/4 L	0 L	1/4 L	1/2 L	3/4 L
Root exposure depth	0 cm	2 cm	3 cm	5 cm	0 cm	2 cm	4 cm	6 cm
Change in plant height (cm)	1.4 ± 0.2a	0.4 ± 0.2b	0.3 ± 0.1b	0.6 ± 0.1b	0.3 ± 0.1a	0.5 ± 0.1a	0.4 ± 0.1a	0.5 ± 0.2a
Plant height range (%)	47 ± 9	12 ± 5	9 ± 3	18 ± 2	10 ± 2	19 ± 4	12 ± 4	14 ± 6
Variation of leaf blade number (slice/basin)	−6 ± 9a	1 ± 8a	18 ± 24a	−2 ± 14a	5 ± 2a	−2 ± 4a	2 ± 7a	−6 ± 14a
Leaf blade number variation (%)	−11 ± 20	1 ± 28	39 ± 53	7 ± 47	19 ± 9	−7 ± 13	3 ± 12	−5 ± 25
Survival rate (%)	88 ± 17a	89 ± 10a	79 ± 11a	87 ± 6a	100 ± 0a	94 ± 7a	91 ± 8a	84 ± 17a

**Note:**

Values were means of three replicates ± standard error, different lowercase letters indicated significant difference (*P* < 0.05).

### Sample collection and processing

On January 15, 2019, the roots of *H. scoparium* and *C. korshinskii* were exposed after the seedlings emerged. Three bags of *H. scoparium* and *C. korshinskii* were randomly selected before treatment. The seedling bags were peeled off, then the plants and sand were poured into the water. After washing, 10 seedlings were taken out to measure the root length, calculate the average root length (L), and then the root exposure treatments were divided into four gradients according to the average root length, named CK (0 L), 1/4 L, 1/2 L, 3/4 L, 20 pots were needed for each treatment group, from which three banks, labeled A, B, and C was selected immediately to measure plant height, leaf number and plant number before and after treatment. On the third day after treatment, plant height, leaf blade number, and plant number of A, B, and C were measured on each treatment group.

### Determination of photosynthetic indexes

Stomatal conductance (Gs), intercellular CO_2_ (Ci), transpiration rate (Tr), and photosynthetic rate (Pn) of the selected *H. scoparium* and *C. korshinskii* were measured using the TARGAS-1 portable photosynthetic measurement system. Each treatment was calculated for three replicates, and the leaf blade area of each treatment was measured with a leaf blade area scanner for later conversion of photosynthetic indexes. Another three of the 20 treated pots were put into foam boxes and brought back to the laboratory. The measurement of root activity and membrane permeability was completed on the afternoon of the test day. The leaves of the remaining 17 test pots were quickly wrapped with foil and marked, put into a vacuum flask filled with liquid nitrogen, and brought back to the laboratory for other physiological indicators.

The transpiration rate (Tr), stomatal conductance (Gs), photosynthetic rate (Pn), intercellular CO_2_ concentration, and photosynthetic index of plants were measured by TARGAS-1 (PP Systems) at 9:00–11:00 am on the 3rd day after treatment. Three bags of each plant were selected, and three leaves were randomly chosen for each treatment. Each leaf was measured twice.

### Determination of physiological indexes

After saving the data, the leaves were put into a self-sealing bag and taken back to the laboratory with a number. The content of chlorophyll was measured by spectrophotometry (Porra, Thompson & Kriedemann, 1989), the content of proline was measured by ninhydrin method (Bates, Waldren & Teare, 1973), the content of soluble sugar was measured by anthrone method (Yemm & Willis, 1954), the activity of peroxidase (POD) was measured by guaiacol method (Hemeda & Klein, 1990), the movement of SOD was measured by NBT reduction method (Mostofa, Hossain & Fujita, 2015), the activity of catalase (CAT) was measured by the ultraviolet absorption method (Hossain, Hasanuzzaman & Fujita, 2010), and the root activity was measured by TTC staining method (Wang, 2006). Ion permeability (membrane permeability) was measured by the conductance method (Wang, 2006), the content of malondialdehyde (MDA) was determined by thiobarbituric acid method (Heath & Packer, 1968).

## Results and Discussion

The changes of growth indexes of *H. scoparium* and *C. korshinskii* under different root exposure treatments are shown in Table 1. Compared with the control group, only the increase of plant height was significantly lower in the treatment group (*P* < 0.05). The number of plants, number of leaves, and various parameters of survival rate were not significantly different from those in the control group after 3 days. There were no significant differences in plant height, plant number, leaf blade number, and survival rate between the treatment group and the control group of *C. korshinskii*, but the survival rate of *C. korshinskii* decreased gradually with the increase of root exposure depth after 3 days.

### Effect of root exposure on the Malondialdehyde (MDA) and Membrane permeability (MP) of *H. scoparium* and *C. korshinskii*

**Figure 1:**
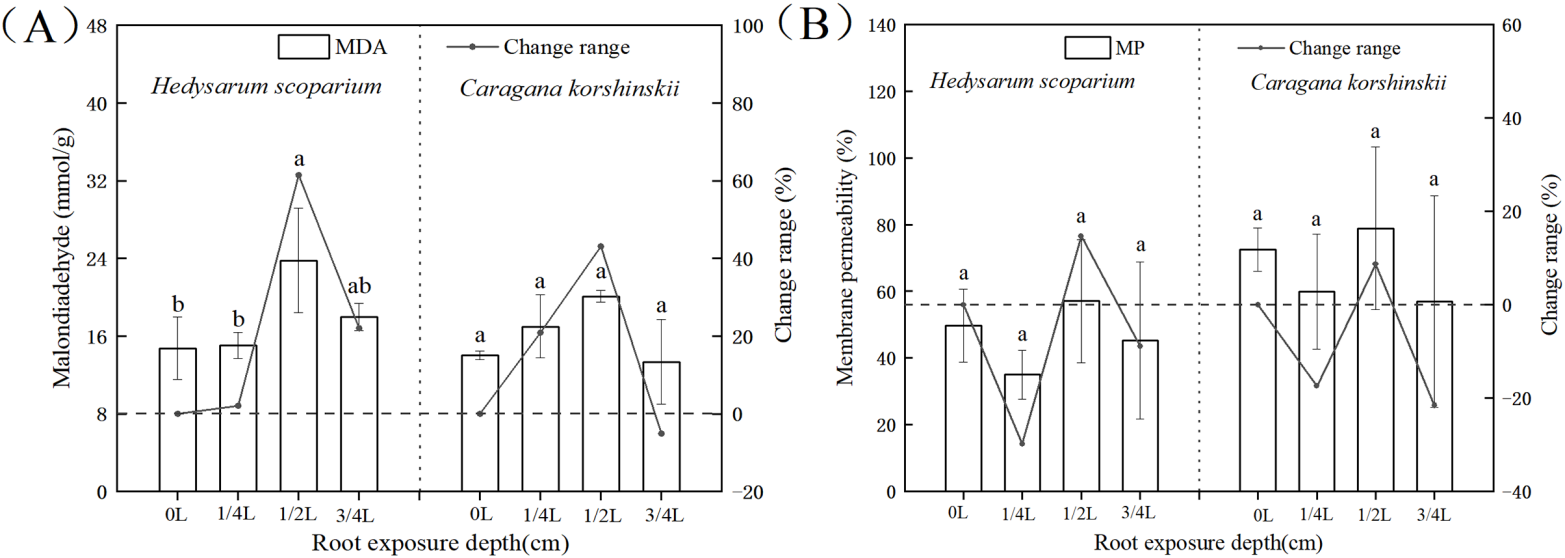
Effect of root exposure on the Malondialdehyde (MDA) (A) and membrane permeability (MP) (B) of *Hedysarum scoparium* and *Caragana korshinskii*. (A) The effect of root exposure on the Malondialdehyde (MDA) of *Hedysarum scoparium* and *Caragana korshinskii*. (B) The effect of root exposure on the Membrane permeability (MP) of *Hedysarum scoparium* and *Caragana korshinskii*. The results were expressed as mean +/− SD, *n* = 3. There are significant differences between different letters.

As shown in Fig. 1A, under the root exposure treatments of the two plants, the MDA content increased first and then decreased with the increase of root exposure depth. Under the 1/4 L root exposure treatment, the MDA content increased slowly. Under the 1/2 L root exposure treatment, the MDA content reached the maximum, and there was a significant difference with the control group (*P* < 0.05).

Cell membranes played a significant role in regulating and controlling plant material exchange and compartmentalization (Lucas et al., 2013; Heidarvand & Amiri, 2010). MDA is the product of membrane lipid peroxidation after plants are subjected to stress. Its production can aggravate membrane damage. The degree of membrane lipid peroxidation can be understood by understanding MDA, so as to indirectly determine the degree of damage to the membrane system (Xie, Zhang & Liu, 2010). Under the 3/4 L root exposure treatment, the MDA content showed a downward trend, indicating that the cell membrane had been basically disintegrated under this treatment, and the plants had reached the endangered state, leading to the production of non-peroxide products.

Membrane permeability is a manifestation of the degree of damage to cells. Usually the lower the membrane permeability, the more complete the membrane structure. As shown in Fig. 1B, under the root exposure treatments of the two plants, the membrane permeability first decreased, then increased and finally decreased with the increase of root exposure depth. Under 1/4 L root exposure treatment, the membrane permeability decreased, which may be due to the initiation of some stress resistance mechanism in plants, thus protecting the structure of the cell membrane. Under 1/2 L root exposure treatment, with the increase of root exposure depth, this stress resistance mechanism has been unable to resist adversity, so that the membrane permeability has increased. Under 3/4 L root exposure treatment, the cell membrane of the two plants may have been damaged, resulting in inaccurate results of the measured membrane permeability, and thus a decreasing trend.

### Effects of root exposure on the photosynthetic index of *H. scoparium* and *C. korshinskii*

**Figure 2:**
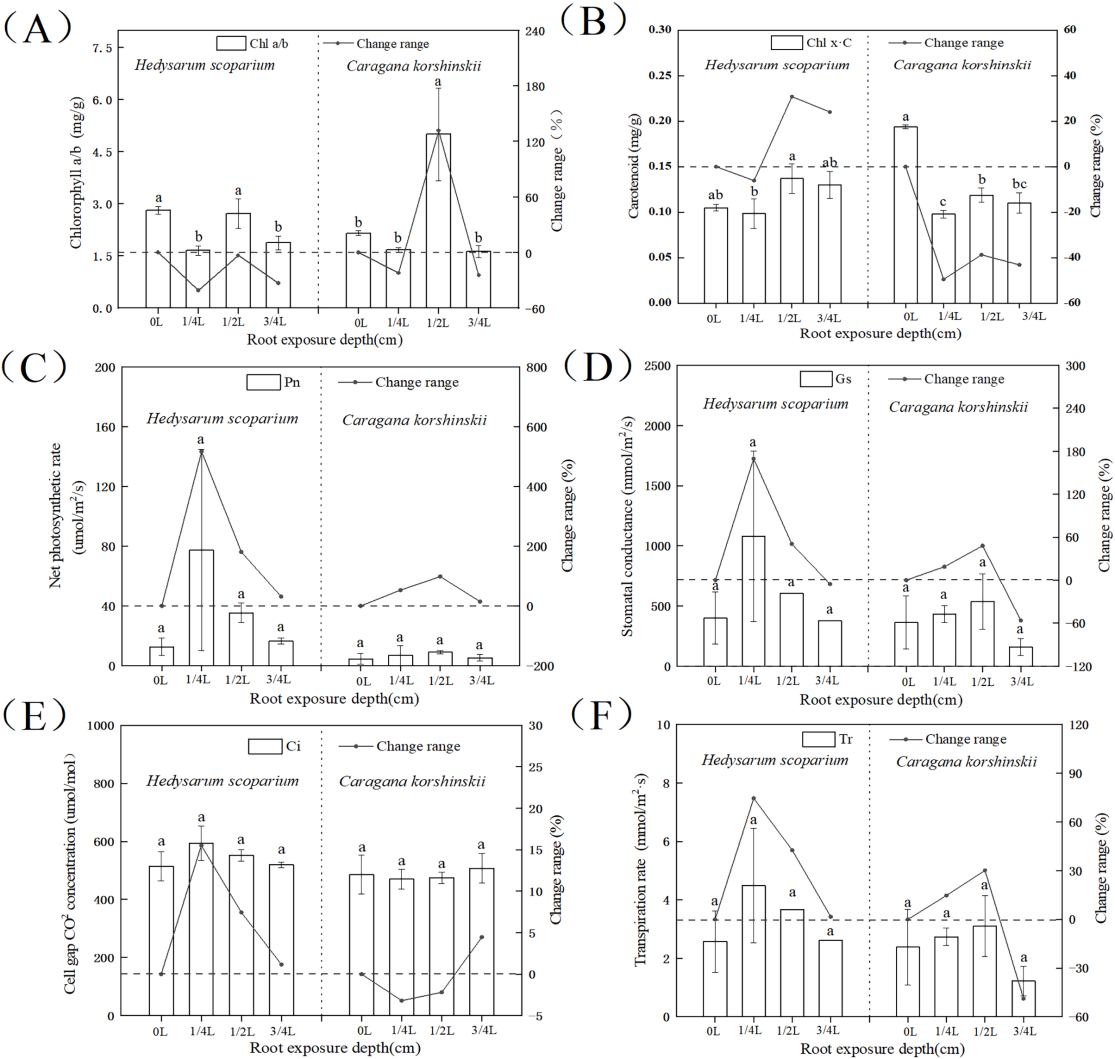
Effects of root exposure on chlorophyll ab (A), carotene (B), net photosynthetic rate (C), intercellular CO_2_ concentration (D), stomatal conductance (E) and transpiration rate (F) of *Hedysarum scoparium* and *Caragana korshinskii*. (A) The effect of root exposure on the chlorophyll a/b of *Hedysarum scoparium* and *Caragana korshinskii*. (B) The effect of root exposure on the carotene of *Hedysarum scoparium* and *Caragana korshinskii*. (C) The effect of root exposure on the net photosynthetic rate of *Hedysarum scoparium* and *Caragana korshinskii*. (D) The effect of root exposure on the intercellular CO_2_ concentration of *Hedysarum scoparium* and *Caragana korshinskii*. (E) The effect of root exposure on the stomatal conductance of *Hedysarum scoparium* and *Caragana korshinskii*. (F) The effect of root exposure on the transpiration rate of *Hedysarum scoparium* and *Caragana korshinskii*. The results were expressed as mean +/− SD, *n* = 3. There are significant differences between different letters.

The higher the ratio of chlorophyll a/b, the stronger the drought resistance. As shown in Fig. 2A, the chlorophyll a/b content under three root exposure treatments was lower than that under CK, while the chlorophyll a/b content under 1/2 L root exposure treatment was significantly higher than the CK (*P* < 0.05). It indicated that root exposure was not conducive to drought tolerance of *H. scoparium*, while *C. korshinskii* was the opposite. With the increase of root exposure depth, the carotenoid content in *H. scoparium* (Fig. 2B) showed an overall upward trend, while the carotenoid content in *C. korshinskii* showed an overall downward trend. It indicated that under root exposure treatment, *H. scoparium* adapted to stress environment by regulating the antioxidant regulation mechanism of carotenoids. *C. korshinskii* resists stress by regulating chlorophyll.

The greater the stomatal conductance, the more carbon dioxide comes in, and the photosynthetic rate will increase. Under the three root exposure treatments, the net photosynthetic rate (Fig. 2C), intercellular CO_2_ concentration (Fig. 2D), stomatal conductance (Fig. 2E), and transpiration rate (Fig. 2F) were all increased compared with those of CK in *H. scoparium*, which were the most obvious under 1/4 L root exposure treatment. There was no significant change in *C. korshinskii* (stomatal conductance and transpiration rate decreased under 3/4 L root exposure, but plants were in an endangered state under the root exposure depth, and they could not control the opening and closing of stomata).

### Effects of root exposure on the Root activity of *H. scoparium* and *C. korshinskii*

**Figure 3:**
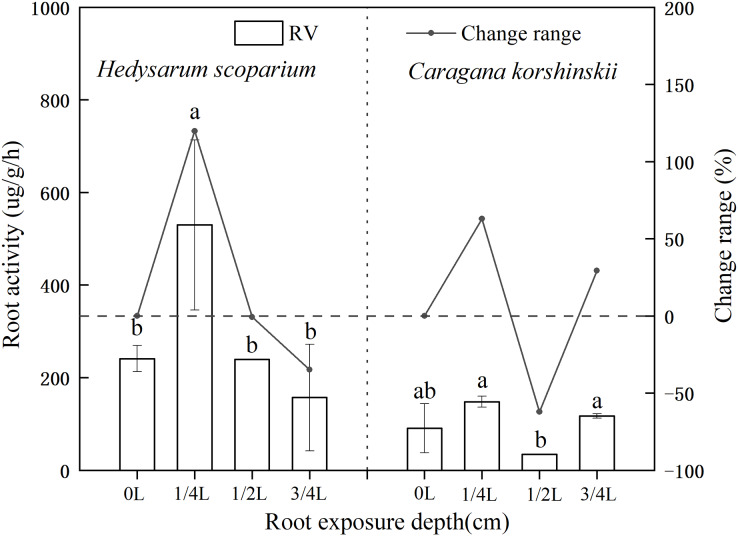
Effect of root exposure on the Root activity of *Hedysarum scoparium* and *Caragana korshinskii*. The figure shows the effect of root exposure on the chlorophyll a/b of *Hedysarum scoparium* and *Caragana korshinskii*. The results were expressed as mean +/− SD, *n* = 3. There are significant differences between different letters.

Root activity generally refers to the absorption, synthesis, oxidation and reduction ability of roots, which is a physiological index that objectively reflects root life activities (Zhang et al., 2019; Haixin, 2020). As shown in Fig. 3, the root activity of *H. scoparium* increased at first and then decreased under different root exposure treatments. Under 1/4 L root depth exposure, the root activity reached the maximum value by regulating its own respiration rate, which was significantly higher than that of the control (*P* < 0.05). However, under 1/2 L and 3/4 L root exposure, the *H. scoparium* may be unable to resist stress by this way because of its dying state. With the increase of stress, the activity of plant roots increased first and then decreased. The increase was due to the emergency response of plant roots to environmental changes. To a certain extent, the decrease was due to the serious damage of plant self-regulation function. Root activity is measured by the total activity of all dehydrogenases responsible for root respiration. Root activity is strong, representing strong root respiration, which can better absorb water and nutrients. The root activity of *C. korshinskii* had no significant change compared with the control under three exposure depths, and the root activity of *C. korshinskii* was lower than that of the whole *H. scoparium*.

### Effects of root exposure on osmotic adjustment substances of *H. scoparium* and *C. korshinskii*

**Figure 4:**
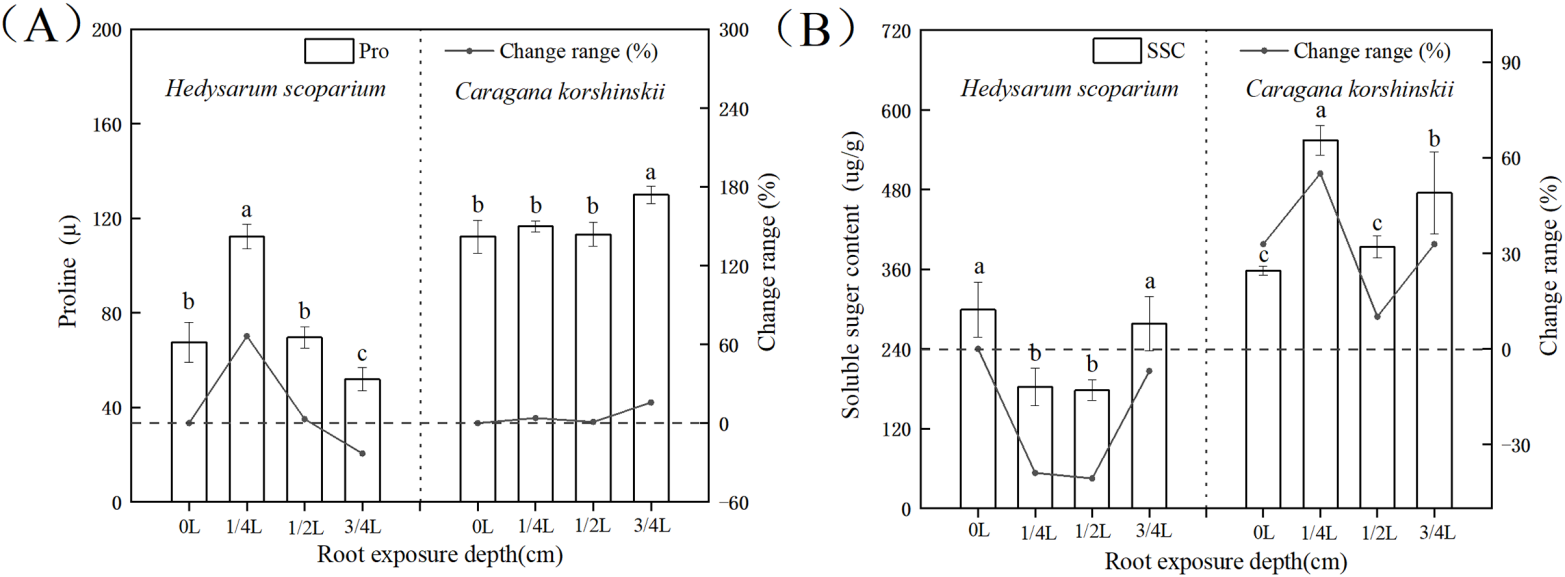
Effects of root exposure on proline (A) and soluble sugar content (B) of *Hedysarum scoparium* and *Caragana korshinskii*. (A) The effect of root exposure on the proline of *Hedysarum scoparium* and *Caragana korshinskii*. (B) The effect of root exposure on the soluble sugar content of *Hedysarum scoparium* and *Caragana korshinskii*. The results were expressed as mean +/− SD, *n* = 3. There are significant differences between different letters.

In plant-cells, proline and soluble sugar, as osmotic regulating substances, could not only reflect the degree of environmental stress on plants, but also serve as the indicators for plant stress resistance. Plants with strong resistance to stress would maintain a high level of osmotic regulatory substances, regulate cell osmotic potential, and protect cell membranes (Gholami Zali & Ehsanzadeh, 2018; Ghaffari et al., 2019, 2021; Shamsul et al., 2012). Figure 4A showed that with the deepening of root exposure depth, the proline content of *H. scoparium* increased first and then decreased. Under 1/4 L root exposure treatment, the proline content reached the maximum, and was significantly higher than that of CK (*P* < 0.05). The proline content of *C. korshinskii* increased significantly only under 3/4 L root exposure, and the proline content increased at this time, because the plants showed an endangered state, a large number of proteins in the body had been decomposed, and free proline was released. However, this osmotic adjustment function was still unable to maintain its normal physiological function.

In summary, from the perspective of osmotic adjustment substances, both plants increase juice concentration by increasing some solute, resulting in a decrease in water potential. *H. scoparium* mainly reduced its water potential by regulating proline concentration; *C. korshinskii* mainly reduces its water potential by regulating soluble sugar concentration to adapt to environmental stress caused by root exposure.

### Effects of root exposure on Antioxidant Protective Enzymes of *H. scoparium* and *C. korshinskii*

**Figure 5:**
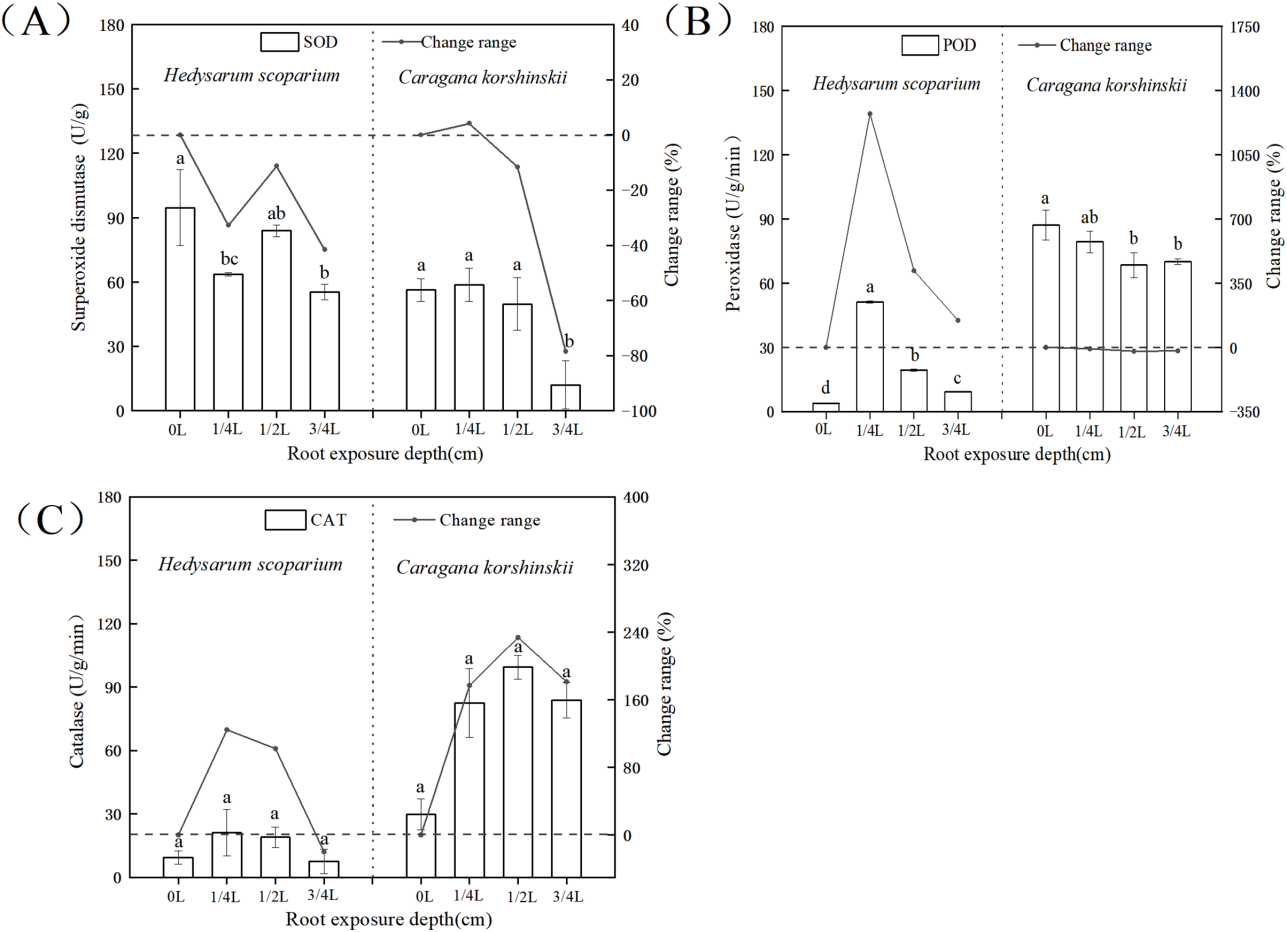
Effects of root exposure on superoxide dismutase (A) peroxidase (B) and catalase (C) of *Hedysarum scoparium* and *Caragana korshinskii*. (A) The effect of root exposure on the superoxide dismutase of *Hedysarum scoparium* and *Caragana korshinskii*. (B) The effect of root exposure on the peroxidase of *Hedysarum scoparium* and *Caragana korshinskii*. (C) The effect of root exposure on the catalase of *Hedysarum scoparium* and *Caragana korshinskii*. The results were expressed as mean +/− SD, *n* = 3. There are significant differences between different letters.

In the antioxidant enzyme system, superoxide dismutase could catalyze the conversion of oxygen free radicals into H_2_O_2_. At the same time, peroxidase and catalase decomposed the H_2_O_2_ produced by the action of superoxide dismutase together to generate harmless H_2_O_2_, the coordination of the three enzymes could effectively eliminate the activity generated in the metabolic process (Shah & Nahakpam, 2012; Gholami Zali & Ehsanzadeh, 2018; Farooq et al., 2009). As shown in Fig. 5A, there was no significant increase in SOD activity in both *H. scoparium* and *C. korshinskii*, indicating that no superoxide anion was produced in the two plants with the increase of root exposure depth. Therefore, plants did not initiate SOD antioxidant protection mechanism. The POD of the control and each treatment group (Fig. 5B) of *H. scoparium* was 1/4 L > 1/2 L > 3/4 L > 0 L, and the three treatments increased by 1,272%, 417% and 146% compared with the control (*P* < 0.05). With the increase of root exposure depth, POD of *C. korshinskii* showed a downward trend. Therefore, *C. korshinskii* did not initiate POD antioxidant protection mechanism. With the increase of root exposure depth, the CAT activity of the two plants showed an increasing trend compared with CK, and *C. korshinskii* was the most obvious (Fig. 5C). Therefore, under root exposure stress, *C. korshinskii* started CAT antioxidant protection mechanism to remove the rapidly accumulated hydrogen peroxide *in vivo* to adapt to this stress.

In summary, in terms of antioxidant protection mechanism, the degree of enzyme response in the two plants was different, SOD in the two plants did not respond, POD in the *H. scoparium* showed indigenous response, CAT in the *C. korshinskii* showed indigenous response. Therefore, under the root exposure treatment, the *H. scoparium* starts POD regulation control to resist this adversity, while the *C. korshinskii* starts CAT regulation control to resist this adversity.

## Conclusion

Under the same proportion of root exposure, the stress resistance mechanisms of *H. scoparium* and *C. korshinskii* were quite different. *H. scoparium* can resist water stress caused by root exposure by activating peroxidase to remove intracellular peroxides, enhancing root activity and increasing proline content. *C. korshinskii* removes intracellular peroxides by activating catalase, and increases soluble sugar content to reduce water potential to adapt drought stress caused by root exposure. The results of this study can provide reference for improving the survival rate of sowing and aerial seeding afforestation in sandy areas and the later maintenance and management of psammophytes.

## Supplemental Information

10.7717/peerj.14905/supp-1 Supplemental Information 1 Experimental methods.

10.7717/peerj.14905/supp-2 Supplemental Information 2 Physiological response characteristics of *Hedysarum scoparium* and *Caragana korshinskii* seedlings after roots exposure. The original data and statistical results, including the test that was performed, the corresponding test statistic, degrees of freedom, the exact *p*-value and effect size.

## Additional Information and Declarations

### Data Availability

The following information was supplied regarding data availability: The data is available in the Supplemental Files.
