# Supplementary material for: Comparative analysis of physiological responses to environmental stress in Hedysarum scoparium and Caragana korshinskii seedlings due to roots exposure
Source: PeerJ. 2023 Feb 28;11:e14905. doi: 10.7717/peerj.14905 (PMC9983421; doi:10.7717/peerj.14905)
Supplement: Supplemental Information 1 [file peerj-11-14905-s001.docx]

| index | apparatus | reagent | equation | concentration | volume | method |
| --- | --- | --- | --- | --- | --- | --- |
| MDA | 1、spectrophotometer  2、tabletop centrifuge | 1、5%TCA：5g TCA was dissolved in a small amount of distilled water and fixed in 100ml volumetric flask.  2、0.67%（w/v）TBA：Take 0.67 g TBA dissolved in 10 % trichloroacetic acid solution and fixed in 100 ml volumetric flask |  |  |  | thiobarbituric acid  （TCA） |
| MP | conductivity meter |  |  |  |  |  |
| ChLa、ChLb、chlxC | spectrophotometer | 95％ethyl alcohol |  | 95％ | 5ml | spectrophotometry |
| Pn | LI-6400  portable photosynthesis system |  |  |  |  |  |
| Ci | Yaxin-1102Portable photosynthetic transpiration apparatus |  |  |  |  |  |
| Gs | LI-6400  portable photosynthesis system |  |  |  |  |  |
| Tr | LI-6400  portable photosynthesis system |  |  |  |  |  |
| Rv | 1. Spectrophotometer 2. analytical balance | 1、Ethyl acetate (analytical purity)  2、Na2S2O4(analytical purity)powder  3、1％TTC；TTC1.0g, dissolved in a small amount of water, constant volume to100ml. Timely dilution to required concentrations  4、phosphate buffer（1／15mol/L，pH7）。  5、1mol/Lsulphuric acid；55ml concentrated sulfuric acid with a specific gravity of 1.84 was added into the beaker with 500ml distilled water，and diluted to 1000ml after cooling.  6、0.4mol/Lsuccinic acid；succinic acid 4.72g，dissolved in water，constant volume to 100ml. |  |  |  | TTC staining method |
| Pro | 1. spectrophotometer 2. Thermostat water bath 3. analytical balance 4. funnel | 1、Acidic ninhydrin solution : 1.25g ninhydrin was dissolved in30ml acetic acid and20ml6mol / L phosphoric acid ( 32.061ml phosphoric acid added to100ml distilled water ), stirred and heated ( 70 ° C ), stored in the refrigerator.  2、3 % sulfosalicylic acid : 3g sulfosalicylic acid dissolved in distilled water and constant volume100ml.   1. Ice acetic acid 2. Toluene |  |  |  | ninhydrin method |
| SSC | 1、spectrophotometer  2、Thermostat water bath  3、analytical balance  4、oven | 1、Glucose standard solution : 80 °C oven drying to constant weight glucose 100mg, prepared 500ml solution, that is 200μg / ml standard solution.  2、Anthrone reagent : 100mg anthrone dissolved in 100ml dilute sulfuric acid ( 76ml concentrated sulfuric acid and 30ml water ).  3、Alcohol ( 80 % ) : 120ml alcohol dissolved to150ml. |  |  |  | anthrone colorimetry method |
| SOD | 1、spectrophotometer  2、tabletop centrifuge | 1、0.05mol/LPBS，pH7.8：  A mother liquor 0.2mol/LNa_2_HPO_4_·12H_2_O: take Na_2_HPO_4_·12H_2_O（molecular weight  358.14）71.7g；  B mother liquor：0.2mol/LNaH2PO4·2H2O：take NaH_2_PO_4_·2H_2_O（molecular weight 156.01）31.2g。  Volume 1000ml with distilled water  Preparation of 0.05mol / L PBS ( pH7.8 ) : A mother liquor ( Na2HPO4 ) 228.75ml, B mother liquor ( NaH2PO4 ) 21.25ml, with distilled water constant volume to 1000ml.  2、14.5mM methionine solution : 1.0818g Met with phosphate buffer ( pH7.8 ) constant volume to500ml.  3、3mM EDTA-Na_2_：take 0.1117g EDTA-Na_2, The volume was fixed to 100ml with phosphate buffer._  4、60μM riboflavin solution : 0.0023g riboflavin was weighed and stored in phosphate buffer to100ml.  5、2.25mM NBT)：take 0.092g NBTSet the volume to 50 ml with PBS and save in dark. |  |  |  | NBT reduction method |
| POD | 1、spectrophotometer  2、tabletop centrifuge | 0.2mol/Lphosphate buffer  (pH6.0)：  A mother liquor：0.2mol/LNa_2_HPO_4_·12H_2_O: take Na_2_HPO_4_·12H_2_O（molecular weight 358.14）71.7g；  B mother liquor：0.2mol/LNaH_2_PO_4_·2H_2_O：take NaH_2_PO_4_·2H_2_O（molecular weight 156.01）31.2g。  Prepare 0.2mol/Lphosphate buffer  (pH6.0): A mother liquor(Na_2_HPO_4_ )12.3 and B mother liquor(NaH_2_PO_4_ ) 87.7ml Separately  and misce bene to 100ml PBS(0.2M，pH6.0)； |  |  |  | methoxyphenol method |
| CAT | 1、spectrophotometer  2、tabletop centrifuge | 0.15 mol/Lphosphate buffer（pH7.0）： take A mother liquor(Na_2_HPO_4_) 228.75 ml and B mother liquor(NaH_2_PO_4_) 146.25 ml after mixing with distilled water constant volume to500ml. |  |  |  | ultraviolet absorption method |

**soil moisture content**

Soil samples were taken with soil drills, and the weight of soil samples was weighed with a balance of 0.1 g precision, which was recorded as the wet weight M of soil samples. The soil samples were dried for 68 h to constant weight in an oven at 105 °C, and then the dried soil samples were measured, which was recorded as the dry weight Ms of soil samples.

Soil moisture content = ( aluminum box and soil mass before drying − aluminum box and soil mass after drying )/( aluminum box and soil mass after drying − aluminum box mass after drying ) * 100 %
